# A review on machine learning implementation for predicting and optimizing the mechanical behaviour of laminated fiber-reinforced polymer composites

**DOI:** 10.1016/j.heliyon.2024.e33681

**Published:** 2024-06-26

**Authors:** Sherif Samy Sorour, Chahinaz Abdelrahman Saleh, Mostafa Shazly

**Affiliations:** a Department of Mechanical Engineering, The British University in Egypt, El Sherouk City, Cairo, 11837, Egypt; b Department of Mechanical Design and Production Engineering, Faculty of Engineering, Cairo University, 12613, Egypt

**Keywords:** Machine learning, Laminated FRP composites, Mechanical behaviour

## Abstract

The utilization of Machine Learning (ML) techniques in the analysis of the mechanical behavior of fiber-reinforced polymers (FRP) has been increasingly applied in composite materials. The ability to achieve high levels of accuracy, coupled with a reduction in computational cost once the ML models are trained, presents a powerful tool for optimization and in-depth analysis of laminated FRP. This review paper aims to provide insight into the emergence of this trend, offer an overview of various ML algorithms and related subtopics, and demonstrate different implementations of ML from recent studies with a specific focus on the design and optimization of FRP composites. The reviewed studies have exhibited high levels of prediction accuracy and have effectively employed ML to optimize the mechanical properties of composite materials. It was also highlighted that selecting the appropriate ML algorithm and neural network structure is crucial for various problems and data. While the studies reviewed have shown promising results, further research is needed to fully realize the potential of ML in this field.

## 1 Introduction

**Fig. 1:**
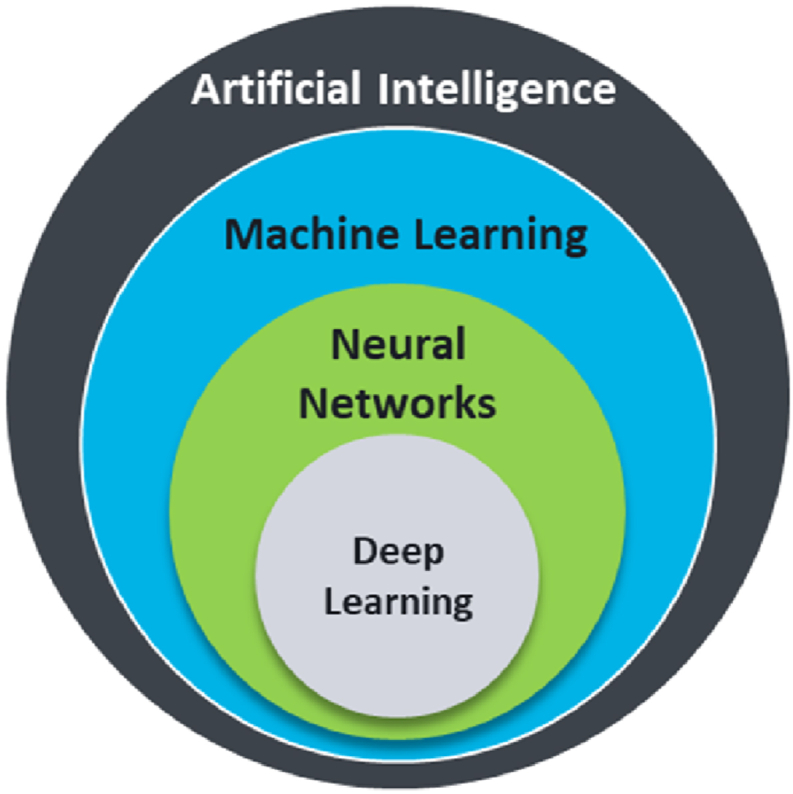
Euler diagram for AI and subfields of ML.

Machine learning (ML), a subset of artificial intelligence (AI), emerged from the early AI developments in the reasoning era between the 1950s and 1970s [1]. AI includes algorithms for tasks requiring human intelligence, whereas ML refers to algorithms that learn from data to perform tasks without explicit programming. ML's subfields include artificial neural networks (ANNs), inspired by neuroscience and structured similarly to the human brain, and deep learning (DL), which involves more complex ANN structures. A graphical representation of the addressed terminologies is represented by the Euler diagram in Fig. 1.

Historically, ML began in 1943 with Walter Pitts and Warren Mcculloch's "Artificial Neural Network" model [2]. The term "AI" was coined by John McCarthy in 1956 [3], and "Machine Learning" by Arthur Samuel in 1959 [4]. Deep learning was first termed by Rina Dechter in 1986 [5]. ML research was mainly theoretical until the 2000s when advancements in computing power, data availability, and open-source libraries like Torch (introduced in 2002) [6] facilitated practical applications. The use of ML in academia has surged, highlighted by significant breakthroughs like DeepMind's AlphaFold in 2021, which predicts protein structures, solving a longstanding challenge [7] and aiding drug discovery and disease understanding. Ongoing advancements, such as MIT's development of neural networks for differential equations in 2022 [8], alongside ML's integration into real-world applications like Google Maps and Facebook's DeepFace, continue to propel the field forward.

The primary aim of this paper is to broaden the readers' understanding of the various implementation techniques where machine learning can be effectively utilized in studies related to the mechanical behavior of laminated composites. This focused review not only highlights existing applications but also identifies new opportunities for innovative research. Given the specialized nature of the topic and the currently limited volume of related literature, this review showcases diverse methodologies and potential enhancements that machine learning can bring to this area of study. Thus, it encourages scholars to explore the potential of ML and apply these advanced techniques in novel ways. In this paper, The second section provides a statistical overview of the current research trend on composites generally and laminated FRP specifically. The third section presents the essential knowledge drawn from our literature review needed to understand the machine learning framework, dataset repositories and generation, and commonly used open-source libraries. The fourth section of the article provides an overview of various research schemes that utilize machine learning, presents a classification of different studies, and a detailed review. The fifth section concludes the paper. An appendix follows, which explores international and institutional collaborations in this field through a network analysis approach, providing additional context and supporting data.

## 2 Preliminary bibliometric analysis

Before conducting a thorough review of laminated FRP composites, a preliminary bibliometric analysis was performed to investigate the utilization of ML in composite materials. The objectives of this study include identifying the beginning of research trends, analyzing the scope of research based on various composite materials, and evaluating worldwide research contributions (Appendix). The analysis is based on articles published in Scopus-indexed journals. The journal selection is based on ten journals specialized in composites and material science and one journal specialized in AI. These journals are; Composite Structures (Elsevier), Composites Part A Applied Science and Manufacturing (Elsevier), Composites Part B Engineering (Elsevier), Composites Science and Technology (Elsevier), Materials Today Proceedings (Elsevier), Polymer Composite (Wiley), Journal of Composite Materials (SAGE), Sensors(MDPI), Polymers (MDPI), Materials (MDPI), and Engineering Applications of Artificial Intelligence (Elsevier).

The keywords used are "Machine Learning" and other related terms like "Artificial intelligence" and subfield keywords like "ANN", combined with keywords of "Composite Materials" and other derivatives like "Composite Structures" and specific terms like "Laminated FRP composite". The time period selected for this analysis was from 2000 to 2022.

### 2.1 Research trend

**Fig. 2:**
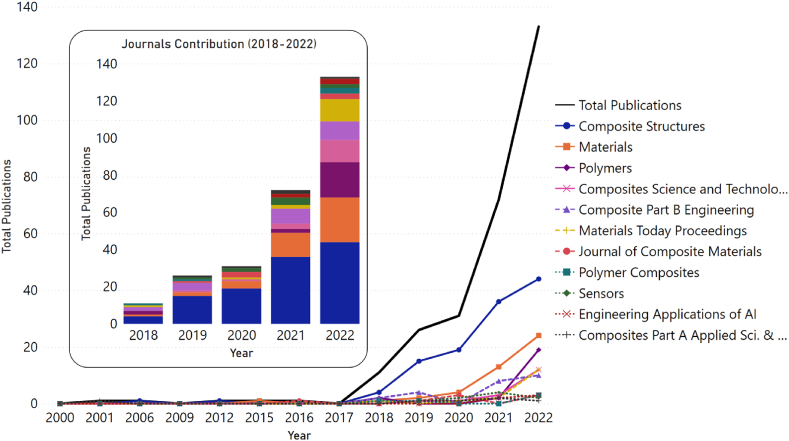
Implementation of machine learning in composite material publications for the period (2000–2022).

After utilizing the specified search criteria and excluding review articles from the results, a total of 278 publications were identified from the year 2000 onwards. As illustrated in Fig. 2, it can be observed that there was a minimal number of publications on an annual basis from 2000 to 2017. Beginning in 2018, there was a noticeable increase in the number of publications, indicating the emergence of a research trend. However, this trend was temporarily disrupted in 2020, possibly due to the COVID-19 pandemic, but has since resumed its exponential growth.

### 2.2 Classified scope based on the composite structure and research objective

**Fig. 3:**
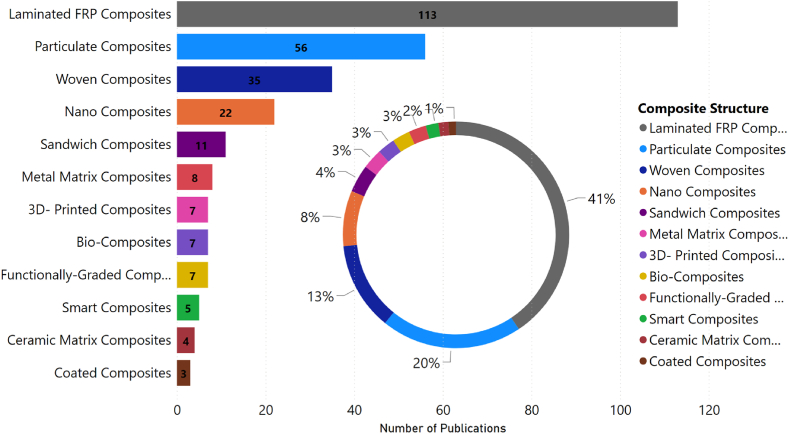
Classified research topics for the period (2000–2022) based on the type of composite structure.

**Fig. 4:**
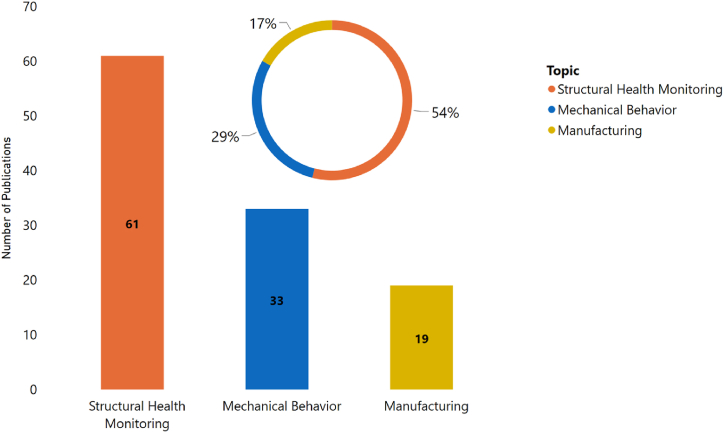
Breakdown of research topics for the period (2000–2022) for laminated FRP composites.

An additional examination of the publications was conducted to categorize the studies according to the composite structure. As depicted in Fig. 3, laminated FRP composites received the most attention in research, accounting for approximately 41 % of the publications. Particulate composites followed this at 20 %, and woven composites at about 13 %. The remaining portion of the research interest was distributed among other structures. A closer examination of laminated FRP composites reveals that the research in this category can be further classified into three subfields, as shown in Fig. 4. Structural health monitoring accounts for 54 % of the research studies, followed by mechanical behavior at 29 %, which would be the focus of our literature review, and manufacturing with the remaining portion.

## 3 Overview of machine learning

In the context of a literature review on the mechanical behavior of laminated FRP composites, this section provides an overview of fundamental background knowledge pertaining to machine learning (ML). The section includes a discussion of relevant algorithms classes and open-source libraries utilized in the literature. Additionally, the section delves into the topic of data repositories and generation as it pertains to ML and its application in the field of laminated FRP composites.

### 3.1 Classification of machine learning models

**Fig. 5:**
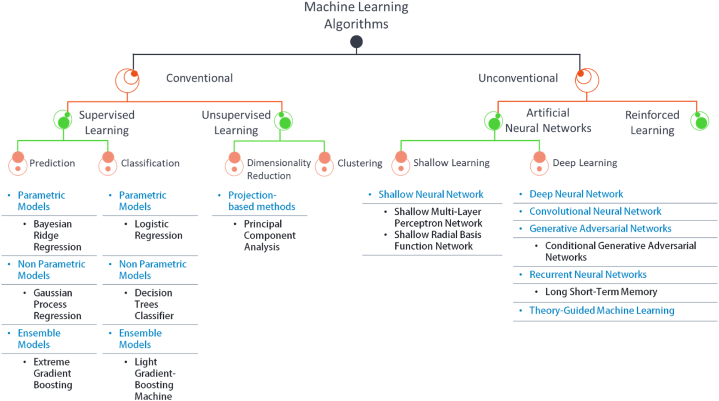
Classification of ML algorithms and examples of some algorithms used in literature.

As illustrated in Fig. 5, Machine Learning (ML) algorithms can be classified into Conventional and Unconventional categories. Conventional ML algorithms are those that have been widely used and well-established in the field. These include supervised and unsupervised learning algorithms. These algorithms are generally simple to interpret and implement and have been successfully applied to a wide range of problems. On the other hand, unconventional ML algorithms are newer methods that have only recently been introduced or are still being researched, such as Theory-Guided Machine Learning. These algorithms often require large amounts of data and computational resources and may be more difficult to interpret and understand, but they can often achieve superior performance on certain tasks. Different classes of ML will be discussed below, with the exception of Clustering and Reinforcement Learning, as they have not been applied in the literature.

#### 3.1.1 Conventional supervised learning

Supervised learning is a type of ML that involves utilizing a labeled dataset to train a model. In other words, the independent and dependent variables are already known in the dataset. Underneath this category are two classes of algorithms for regression (prediction) and classification tasks.

Supervised regression models [9,10] predict a continuous dependent variable based on independent variables, commonly referred to as features. There are several different models, each with its strengths and weaknesses. Parametric models make assumptions about the functional relationship between the independent and dependent variables and use a fixed set of parameters learned from the training data. The simplest model in this category is linear Regression (LR). Another variant of these models is called the Regularization Model, which adds a penalty term to the objective function to prevent overfitting. Overfitting in ML means the model has high prediction accuracy for the dataset used for modeling the function parameters (training) and inconsistent accuracy for any new data (Low Bias/High Variance). Examples of regularization models from the literature include Bayesian Ridge Regression (BR), Least Absolute Shrinkage and Selection Operator (LASSO), and Elastic Net Regression (EN). Non-parametric models do not make any assumptions regarding the relationship between independent and dependent variables. Instead, they employ statistical analysis of the data to identify patterns and make predictions, and they provide the prediction with uncertainty estimates. Such models are robust to outliers and capable of handling nonlinear data. However, they require large datasets to avoid overfitting. Examples of non-parametric models from the literature include Gaussian Process Regression (GPR), Kernel Ridge Regression (KRR), Support Vector Regression (SVR), and Decision Trees (DT). Ensemble models utilize the predictions generated by multiple regression models to enhance overall performance. These models are characterized by their robustness and ability to effectively handle complex nonlinear data, which may pose a challenge for both parametric and non-parametric models. Examples of ensemble models from the literature include Random Forest (RF), Gradient Boosting Regressor (GBR), Classification and Regression Tree (CART), Extreme Gradient Boosting (XGBoost), and Light Gradient-Boosting Machine (LGBM).

The other category of algorithms is supervised classification models [9,10], a class of machine learning models employed to categorize data by identifying the decision boundary separating different data groups. In other words, these models predict a discrete dependent variable based on a set of independent variables through a threshold value determined by the decision boundary. Parametric classification models determine the decision boundary by making assumptions about the functional form between the independent and discrete dependent variables. These models are simple to interpret and perform well on linearly-separable datasets, but they are sensitive to outliers and have limitations on non-linearly-separable data. An example of a parametric model from the literature is Logistic Regression (LR). Non-parametric classification models, unlike Parametric models, rely on identifying geometrical patterns in the data to obtain the decision boundary. These models can handle complex data distribution but at the cost of difficult interpretation of the decision boundary parameters. An example of a non-parametric model from the literature is the Decision Trees Classifier (DTC). Ensemble classification models have the ability to reduce the risk of overfitting and increase robustness. By combining the predictions of multiple simpler models, ensemble models can often achieve a higher level of accuracy. An example of an ensemble model from the literature is the Light Gradient-Boosting Machine (LGBM).

#### 3.1.2 Conventional unsupervised ML algorithms

Unsupervised learning is a type of ML in which the model learns from unlabeled data, meaning the dataset does not have predefined independent or dependent variables. Unsupervised learning aims to discover the underlying structure or pattern in the data. Underneath this category are two classes of algorithms for clustering tasks and dimensionality reduction. From the literature, dimensionality reduction was adopted in conjunction with other ML models. Dimensionality reduction techniques are utilized to decrease the number of variables in a dataset while preserving the maximum possible amount of information. There are multiple models underneath this category, one of which is Projection-based methods. These methods project the data onto a lower-dimensional subspace while preserving specific properties of the data, such as linearity or variance. An example of a Projection-based method from the literature is Principal Component Analysis (PCA).

#### 3.1.3 Artificial neural network

**Fig. 6:**
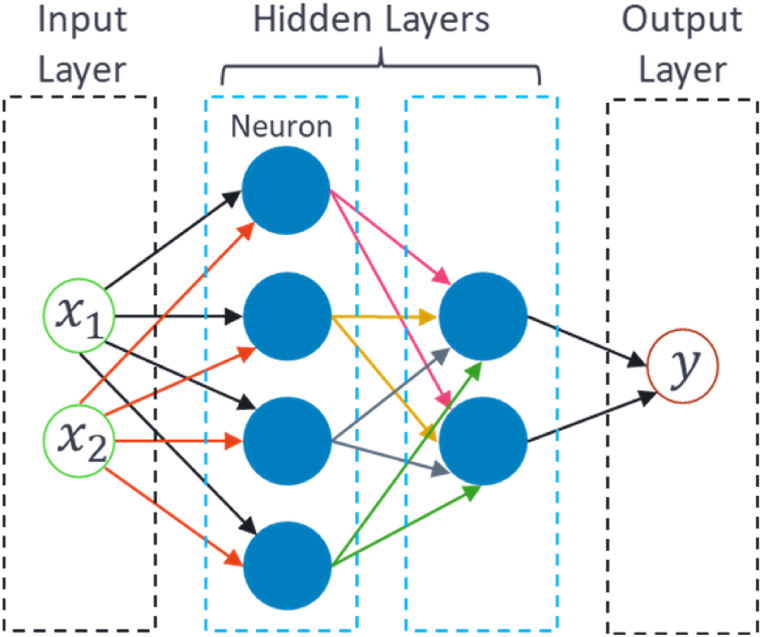
Basic architecture/structure of ANN.

Artificial Neural Networks (ANNs) [[10], [11], [12], [13]] are a class of machine learning models inspired by biological neurons' structure and function. The basic structure of an ANN typically comprises multiple layers, including an input layer, one or more hidden layers, and an output layer. The input layer receives the input data and passes it on to the next layer, while the hidden layers perform complex computations on the input data. The neurons within each layer are interconnected through pathways, referred to as connections, each of which has an associated weight that represents the importance of the input from one neuron to another. Additionally, non-linearity is introduced into the network through activation functions in the neurons, such as sigmoid, ReLU, and tanh. The output layer receives the output from the hidden layers and produces the final output of the network. This framework allows the model to learn more complex relationships between the input and output data, as illustrated in Fig. 6.

Shallow learning refers to the ANN models that learn the data using 1 or 2 hidden layers in the architecture. These models are often called Shallow Artificial Neural Networks (SANN). Radial basis function (RBF) is a type of SANN in which each neuron has a radial basis function as its activation function, and it typically has only one hidden layer. RBF networks are often used for nonlinear regression and classification problems and are faster to train. However, they are only limited to a nonlinear dataset. Another reported type of SANN is a multi-layer perceptron network (MLP), which consists of one or two hidden layers of fully connected feedforward neurons. The neurons' activation functions are often sigmoid, ReLU, and tanh. MLP is a general-purpose network for supervised learning tasks like classification and regression.

Deep learning refers to the ANN models that learn the data using more than two hidden layers in the architecture or have any variations or complexity other than ANN architecture. Based on the literature, the following DL networks were utilized; Deep Neural networks (DNNs) are complex, multi-layered models that can be used for a wide range of tasks, such as image classification, natural language processing, and speech recognition. DNNs are highly expressive models that can learn complex non-linearity of data but also require large amounts of data and computational resources to train.

Convolutional Neural Network (CNN) is a specific type of DNN that is particularly well-suited for image and video processing tasks. They are based on the idea of using convolutional layers to extract features from images and can be used for tasks such as image classification and object detection. CNNs are good at capturing spatial and time-based patterns in the data, but they can be computationally expensive and require a large amount of data.

Long Short-Term Memory (LSTM) network is a subclass of Recurrent Neural Network (RNN) architectures that are specifically designed to handle sequential data. These networks are characterized by the incorporation of memory cells and gates, enabling them to maintain information across prolonged input sequences. However, the main limitation is that it can be computationally expensive and difficult to train, especially for large datasets.

Conditional Generative Adversarial Networks (CGANs) constitute a class of generative models that can be employed to generate new samples from a given input. These models are composed of two primary components: a generator network and a discriminator network, which are trained in a two-player minimax game framework. The generator aims to produce indistinguishable samples from the real dataset, while the discriminator aims to distinguish the generated samples from the real dataset. Through this adversarial process, the generator learns to generate new samples similar to the real dataset.

Theory-Guided Machine Learning (TGML) represents a methodology that integrates machine learning techniques with domain knowledge to guide the development of models. This approach aims to enhance the interpretability and explainability of models, as well as to increase their robustness and generalization performance. The main limitation is that the model is only as good as the quality of the theories that guide it.

### 3.2 Machine learning libraries

A machine learning library is a collection of pre-written code that is specifically designed to perform various tasks related to machine learning, such as data pre-processing, training and evaluating models, and generating predictions. These libraries are written in programming languages such as Python or R, which are preferred in machine learning due to their high-level and general-purpose nature and active community. These libraries are designed to make the process of building machine learning models faster and more efficient for developers and data scientists by allowing them to focus on modeling and problem-solving rather than on the implementation details. The focus in this part will be on open-source Python libraries that represent the core of ML modeling and are reported to be frequently used based on our literature review. These libraries are.

#### 3.2.1 SciKit-learn

Scikit-Learn [14], also known as Sklearn, is a widely-used open-source library for ML that David Cournapeau first developed in 2007 as part of a Google Summer of Code project. Its popularity among data scientists and researchers is attributed to its ease of use and versatility, as it offers a wide range of tools and algorithms for various types of conventional supervised and unsupervised learning. However, it has limited capabilities for ANNs.

#### 3.2.2 Tensorflow – Keras/TensorFlow

TensorFlow [15] was initially released in 2015 by the Google Brain team. Since its release, it has gained widespread adoption in industry and academia. TensorFlow is a DL library designed to build and train neural networks. While TensorFlow is not specifically designed for conventional machine learning, it can be used to implement and train conventional machine learning models. Some popular ANNs implemented in TensorFlow include CNN, CGAN, and LSTM. Because of the interface complexity and difficulty of navigating, Keras [16] is often used in conjunction with TensorFlow. Keras was first released in 2015 by François Chollet, an engineer at Google. It was developed to provide a simplified interface (in technical terms called a wrapper/API) for other DL frameworks.

#### 3.2.3 Pytorch

PyTorch [17] is an open-source DL library that was first released in 2016 by Facebook. It is based on the Torch library and provides similar functionality to TensorFlow. PyTorch is known for its ease of use, and It provides tools for developing new DL models making it a good choice for research and development in the ML field. It also offers various popular neural network architectures, such as CNN, CGAN, and LSTM.

#### 3.2.4 Ensemble models libraries

XGBoost [18] was developed by Tianqi Chen at the University of Washington in 2014. It is a library for ensemble models that are mainly composed of DT. Another ensemble library is LGBM [19], developed by Microsoft and released in 2016. It has similar functionality as XGBoost but with a different approach for structuring DT and better accuracy, speed, and capability for handling large datasets and missing values.

### 3.3 Academic datasets repositories and data generation

#### 3.3.1 Dataset repositories

Academic institutions have collaborated to create data repositories for sharing and collecting datasets in response to the need for sufficient data for machine learning algorithms. These data repositories are designed to make it easy for researchers and data analysts to find and access data sets, thus enabling the use of ML algorithms that requires huge dataset, such as ANN. Several organizations offer persistent identifiers (PIDs) for academic datasets, such as DataCite, to ensure the legitimacy and credibility of these research data. These unique identifiers allow for proper citation of the dataset in publications and incentivize researchers to contribute their data without fear of losing credit. Some examples of open-access repositories and search engines for academic datasets are 4TU.ResearchData (2008) [20], NASA Ames Prognostics Data Repository (2008) [21], Mendeley Data (2015) [22], and Google Dataset Search (2020) [23]. These repositories contain datasets of various disciplines like engineering, science, and medicine, and some of these repositories offer curation services, such as combining and organizing data sets from different sources.

#### 3.3.2 Dataset generation for composite microstructure

Based on our literature survey, one of the alternative tools for dataset generation for composite microstructures images is Python Material Knowledge System (PyMKS) [24]. It is a toolbox package developed by the Center for Hierarchical Materials Design (CHiMaD) at Northwestern University that provides a set of modules for analyzing and modeling complex materials properties and is not limited to composite materials. One of these modules is the microstructure generation module which generates microstructure images with their microscopic properties. There are modules for homogenization to obtain macroscopic properties from the microstructure and modules for feature extraction, such as grain size and orientation statistics. It is a noted-worthy tool that, combined with ML, can further exploit its capabilities.

## 4 Bibliometric review

### 4.1 Research perspective

As ML models offer a variety of tools for mapping complex relationships between data variables, these ML models are trained to map the relationship between selected inputs and output from finite element simulations, experiments, or mathematical models through the training process.

The literature review from an ML perspective reveals two primary areas of investigation. The first area is the development of an ML model tailored to specific tasks, such as predicting the mechanical properties of composite materials. The second area of research is the comparative evaluation of the performance of various ML algorithms, with the goal of identifying the most suitable algorithm for a given application. This evaluation is typically conducted by assessing the algorithms' accuracy, computational efficiency, and robustness (Evaluation metrics). In addition to these primary areas of research, studies in the field of ML may also include supplementary investigations such as correlation analysis to understand the underlying complex relationships between variables and the most significant parameters. Another type of supplementary investigation is by generating large datasets using optimally-trained ML algorithms, followed by an optimization step to determine the optimal design parameters for a given problem.

From the composite material perspective, There are nine main areas of research where ML is implemented. These topics to be discussed are Microstructure-Property linkage, Mechanical properties prediction, Damage parameters calibration, Damage parameters prediction, Damage initiation Prediction, Stiffness degradation analysis, Progressive damage analysis, Crack path detection, and design and optimization of FRP composites.

### 4.2 Machine learning implementations

#### 4.2.1 Microstructure-property linkage

In their study, Kim et al. [25] utilized a CNN to predict the transverse mechanical properties of unidirectional FRP composites from microstructure images. To generate a dataset for training and testing the CNN, the authors randomly generated 900 representative volume element (RVE) models in the microscale, with fiber volume fractions limited to 40 %, 50 %, and 60 %. These RVE models were then converted into binary images and subjected to 900 two-dimensional finite element analyses (FEA) using the cohesive zone model for damage initiation. The resulting dataset consisted of 900 samples, where each sample included a stress-strain curve obtained from FEA and its corresponding binary image of the RVE. The authors reported that the trained CNN achieved high accuracy in predicting the stress-strain curve and, thus, the transverse mechanical properties, given an unseen binary microstructure image.

Similarly, Sengodan [26] proposed a novel ML framework for predicting the stress-strain curve from FRP microstructure images. The framework employed PyMKS to generate 24,000 microstructure images and their corresponding homogenized mechanical properties. The stress-strain curves for uniaxial tensile loading were obtained from Sfepy, a Python-based FEA software. The ML framework first involved transforming the microstructure images and the stress-strain curves through the principal component analysis (PCA) algorithm to decorrelate the data and reduce the computational cost before training a CNN. The framework was reported to be successful in accurately predicting the stress-strain curves, compared to FEA curves, for unseen randomly-generated microstructure images.

Additionally, Bhaduri et al. [27] used a CNN with a U-Net architecture to predict the stress field map from FRP microstructure images. The dataset consisted of 100 samples, where each sample included an image of the microstructure and its corresponding von Mises stress distribution obtained from FEA using RVEs in the microscale under uniaxial transverse loading. The RVE microstructure images represented a random distribution of fibers, with numbers ranging from 6 to 100, and 25 images were generated for each number of fibers. The authors reported that their ML model could predict the von Mises stress field with high accuracy and that the computational cost of using the ML model for prediction was significantly lower than that of traditional FEA software, ABAQUS.

#### 4.2.2 Mechanical properties prediction

Ding et al. [28] employed ML techniques to predict the transverse elastic modulus and transverse tensile strength of unidirectional FRP composites. They conducted 2D FEA using the discrete element method based on an RVE model in the microscale. The design of experiments was used to determine the number of simulations conducted for a range of fiber volume fractions and radii commonly found in unidirectional FRP laminates. As a result, their dataset consisted of 2000 samples that related the fiber volume fraction, fiber radius, transverse elastic modulus, and transverse tensile strength. By training a DNN model with four hidden layers, each containing 80 neurons, the model was able to predict the transverse elastic modulus and transverse tensile strength with an average accuracy (R^2^) of 96.3 % and 96.7 %, respectively, when given the volume fraction and radius of the fibers.

Within a similar scope of interest, Zhenchao et al. [29] utilized ML to predict the elastic properties of carbon fibers from cross-ply carbon fiber-reinforced polymer (CFRP) elastic properties. They conducted 3D FEA based on a simplified mesoscale RVE model and established a dataset for CFRP micro and macro properties. They employed the CART algorithm for the prediction of longitudinal and transverse elastic modulus and shear modulus of the carbon fibers. It was noticed that the depth of the tree that resulted in high prediction metrics varied depending on the type of carbon fiber property, as discussed in their publication in detail.

#### 4.2.3 Damage parameters determination

##### 4.2.3.1 Parameters calibration

In a study published by Freed et al. [30], a methodology was proposed for determining the failure parameters of the Virtual Crack Closure Technique (VCCT) for adhesively bonded composite joints through the use of the ASTM Mixed Mode Bending (MMB) test. The motivation behind this study stems from the limitations of the current ASTM standards, which only apply to laminated composites and do not consider bonded composites. To address this, an interval of expected values of VCCT was assumed, and a database was established utilizing 5632 2D FEA simulations of the MMB test and spanning these parameters. The GPR algorithm was selected to optimize the parameters in relation to the 88 MMB test experiments conducted, with the goal of minimizing the error of the failure load value obtained from FEA compared to the experimental value. The resulting model was able to predict the VCCT parameters with high accuracy successfully. Furthermore, the study highlighted that the fracture energies calculations from ASTM standards are not valid for adhesively bonded composites.

Similarly, Yuan et al. [31] introduced an alternative method for determining cohesive parameters from microdroplet experiments through the use of ML techniques rather than relying on traditional analytical methods. This approach involved assembling a dataset through the conduct of 120 experiments and 12,768 2D FEA simulations utilizing cohesive elements at the fiber-resin interface. The ML approach consisted of two steps; first, the LASSO algorithm was utilized for feature selection, followed by the use of the KRR algorithm for prediction. The resulting model demonstrated accuracy in predicting interfacial shear strength and shear fracture toughness comparable to conventional methods.

##### 4.2.3.2 Parameters predictions

In their study, Karamov et al. [32] examined the potential for predicting the fracture toughness of unidirectional FRP composites through the utilization of various mechanical properties, such as tensile strength and modulus. The dataset for this study was comprised of 900 samples collected through the conduct of 650 standardized experiments. Five different ML algorithms were selected and compared for their prediction accuracy, including RF, XGBoost, SVR, GPR, and a DNN with three hidden layers and (20,40,10) neurons, respectively. The RF and XGBoost algorithms were the most accurate, with an error of approximately 10 % from the mean fracture toughness value. A further investigation was also conducted using these two algorithms, which revealed a correlation between longitudinal compression modulus and tensile strength with fracture toughness, as previously noted in other publications.

Yin et al. [33] performed a comparative study on various ML algorithms for the purpose of identifying the most accurate algorithms for predicting the interfacial shear strength (IFSS) of fiber-matrix interfaces and maximum load from pullout tests. The dataset for this study was collected from 12 publications that conducted pullout experiments and simulations, resulting in a total of 922 samples (818 experiments and 104 FEA). The selected ML models included LASSO, BR, EN, SVR, GBR, and SANN-MLP (with two hidden layers of 15 neurons each). Based on evaluation metrics for regression analysis, the GBR algorithm and the SANN outperformed the other algorithms, with GBR performing slightly better than SANN. Both GBR and SANN were able to accurately predict the IFSS, but with lower prediction accuracy for the maximum load. An additional analysis based on the GBR relative importance score for interfacial shear strength also revealed that decreasing fiber diameters has the most significant influence on increasing the IFSS.

In 2020, Zobeiry et al. [34] employed ML as an alternative method for conducting over-height compact tension (OCT) experiments on quasi-isotropic CFRP composites. This study aimed to predict the fracture energy and strain-softening curve that would be obtained experimentally. Using LS-DYNA and an isotropic coupled damage-plasticity material model, 10,000 FEA (2D) simulations were conducted to simulate the experiment. The extracted parameters from the load-displacement curve and key material properties were collected and structured as 10,000 samples for training a DL model, specifically a TGML network, which comprises 4 neural networks in series, each with 4 hidden layers of 10 neurons. The predicted load-displacement behavior was reported to match well with that of the OCT experiments. In 2021, the same authors [35] employed ML for the same purpose but for conducting compression fracture experiments utilizing the LSTM network. They conducted 15,000 FEA (2D) simulations of compact compression tests, and the resulting output load-displacement data points and other key features (such as material properties and maximum load) were collected and structured as 15,000 samples. The LSTM model was able to accurately predict the load-displacement graphs, and the authors noted that a minimum of 5000 samples is required for this type of ML model and problem.

In a different application of ML, Cidade et al. [36] proposed a new technique based on digital image correlation to determine mode I dynamic fracture toughness for laminated FRP composites from the Split-Hopkinson Pressure Bar experiment. The role of ML in this study was to determine the relevant importance of the input variables in calculating the modified J-integral.

Barshan Dev et al. [37] present an extensive study that explores the role of ML algorithms in predicting the properties of reinforced composites, emphasizing the critical nature of statistical indexes in these predictions. This paper conducts a methodical review and comparison of diverse ML models that predict mechanical, thermal, tribological, acoustic, and electrical properties across various types of composites. The dataset was assembled from numerous studies. The key ML algorithms discussed include artificial neural networks (ANN), support vector machines (SVM), and decision trees (DT), among others. The core findings reveal varied efficacy of ML models based on the property being predicted, highlighting the importance of choosing an appropriate model tailored to the property of interest. This investigation not only deepens the understanding of ML applications in materials science but also sets a benchmark for future studies to refine prediction models further, enhance data collection and preprocessing techniques, and explore innovative composite materials applications.

Tobias Würth et al. [38] leverage physics-informed neural networks (PINNs) to optimize the thermochemical curing processes of composite materials. The dataset, crafted through sophisticated mathematical modeling of the curing process, allows PINNs to train effectively without the extensive data typically required. This approach generates data over 500 times faster than traditional finite element simulations, dramatically expediting the optimization process, and significantly expediting the process optimization. By incorporating physical laws directly into the learning algorithms, the PINNs ensure that the outcomes are not only efficient but also adhere to physical realities, enhancing the reliability of predictions related to the mechanical properties of composites. This methodology enables precise control over manufacturing parameters, thereby directly affecting the strength, durability, and overall mechanical properties of the final product.

#### 4.2.4 Damage initiation

##### 4.2.4.1 Prediction of damage initiation

Zhang et al. [39] conducted a comparative study to evaluate the performance of DNN (3 hidden layers of 30 neurons each) and RF for predicting failure indexes in composite laminates under in-plane stress loading, open-hole laminate under uniaxial tension and critical buckling eigenvalues of open-hole laminate under uniaxial compression. The study employed FEA (2D) with a total of 10,000, 13,715, and 13,917 simulations for the mentioned cases, respectively. The study results indicate that DNN performed better than RF in prediction accuracy, while RF demonstrated improved computational efficiency.

Li et al. [40] presented a novel deep transfer learning (TL) methodology aimed at constructing the Allowable Load Space (ALS) of notched composite laminates by predicting the damage initiation loads of notched laminates under varying design parameters such as geometry, material properties, and loading conditions. The randomly generated dataset was of 36,000 samples FEA simulations, capturing a broad range of mechanical behaviors across different laminate configurations. This dataset was used first to train an ensemble deep neural network to capture the interactions between various design parameters with a high accuracy for known design parameters. The ML model is then fine-tuned using 30 samples of new design parameters, transforming it into an Ensemble-TL model. This ensemble-TL model demonstrates the ability to generalize to new design spaces, making it a powerful tool for predicting allowable loads under varied and new design conditions. The constructed ALS enabled direct determination of optimal laminate configurations for given load conditions, dramatically reducing the design iteration time and resource expenditure typically required in composite structure design.

##### 4.2.4.2 Computational costs reduction

The work of Post et al. [41] examined the use of machine learning (ML) to improve the computational efficiency of damage initiation criteria based on the Mohr-Columb criteria. These criteria, such as LaRC and Puck's criteria which are based on Mohr-Columb criteria, have been shown to provide more accurate predictions of compressive failure behavior in composites than Hashin's criterion. However, they are computationally expensive due to the need for iterative searches for matrix crack angles. To address this issue, the authors employed a two-step methodology involving both classification and regression ML models. The first step utilized LogR, LGBM, and ANN models to predict damage initiation through occurrence classification. The second step employed LR, LGBM, and ANN models to predict the crack angle. The performance of the models was evaluated based on a combination of 10,000 stress-state variables that satisfied the Mohr-Columb criteria for both plane stress and 3D stress states. After conducting a comparative study, the authors found that the ANN was the best algorithm for both steps in the methodology, with the adopted SANN-MLP comprising two hidden layers of 16 neurons for the plane stress problem and DNN comprising 5 hidden layers of 128 neurons each for the 3D stress state problem.

#### 4.2.5 Stiffness degradation analysis

##### 4.2.5.1 Laminated composite confined cracks under in-plane stress loading

Yuan et al. [42] conducted a study in which Machine Learning (ML) techniques were applied to predict the stiffness degradation of cross-ply CFRP laminates due to matrix cracks. The dataset utilized in the study consisted of 7000 samples, which were obtained from a series of FEA simulations performed using a 3D mesoscale RVE model that confined central cracks. The ML algorithm employed in the study was the SANN-MLP model, which consisted of two hidden layers of 50 neurons each. The study's results indicated that the proposed ML model could accurately predict the equivalent stiffness of the laminates, with an average accuracy (R^2^) of 95 %.

##### 4.2.5.2 Laminated composite confined cracks under uniaxial tensile loading

In their publication, Yuan et al. [43] proposed an alternative approach for predicting the stiffness degradation in cross-ply CFRP and GFRP laminates under uniaxial tensile loading. A dataset of 467 samples was gathered from 14 publications that conducted either experimental work or FEA (2D/3D) for both CFRP and GFRP. The proposed approach employed a two-step ML methodology, utilizing the LASSO algorithm for feature selection and the KRR algorithm for prediction. The model achieved an average accuracy (R^2^) of 95 % for predicting the normalized axial modulus of the laminates. These two studies by Yuan et al. [42,43] demonstrate the versatility of ML approaches and the importance of selecting the appropriate methodology based on the size and nature of the available data.

#### 4.2.6 Progressive damage analysis

##### 4.2.6.1 Laminated composite confined circular hole under in-plane loading

Zhang et al. [44] developed a predictive model utilizing ML techniques to capture the progressive damage behavior of CFRP laminates with a circular hole. A dataset was generated through the use of 3D FEA based on the mesoscale representative unit cell (RUC) model. A comprehensive comparison of various DNN network structures determined that a DNN model comprising of three hidden layers, each containing 80, 60, and 70 neurons, respectively, could successfully predict the damage initiation. However, when predicting the true stress-strain curves, the model demonstrated high consistency under uniaxial tension and pure shear loading conditions, while the prediction accuracy was relatively lower for other loading conditions.

Reiner et al. [45] innovatively combined 2D FEA, machine learning (ML), and Markov Chain Monte Carlo (MCMC) methods to incorporate uncertainty into progressive damage simulations of fiber-reinforced polymers (FRPs). The study conducts 15,000 FEA simulations of open-hole tension tests on FRP composites using varied input parameters, creating a large dataset that trains a neural network with five hidden layers to develop an efficient surrogate model. This model is crucial for executing Bayesian parameter estimation via MCMC, allowing for the statistical estimation of FEA input parameters' probability densities. The research addresses inherent uncertainties in mechanical properties due to variations in manufacturing processes and material defects. By accurately predicting mechanical behaviors before manufacturing and physical testing, this approach significantly reduces cost and time, enhancing the predictive accuracy of composite performance under uncertain conditions.

##### 4.2.6.2 Ply drop laminated composite under in-plane displacements

Mendoza et al. [46] developed a novel strategy for selecting the appropriate ANN structure to model the progressive damage behavior in composite materials with ply drop geometries. The approach is based on a deeper understanding of the internal workings of ANNs and the utilization of the Greedy Neural Network Search (GNAS) algorithm to identify the optimal network structure. The dataset employed for this study consisted of 356,273 samples, which were obtained from 396,800 FEA simulations using a 2D Linear Perturbation Analysis (stiffness degradation) and Surface-Based Cohesive Behavior approach (damage initiation). Through multiple investigations, the authors found that a deep neural network (DNN) model, consisting of 7 hidden layers of (9, 77, 78, 82, 89, 9, 1) neurons each, yielded the highest prediction accuracy for the progression of damage in ply drop composites.

##### 4.2.6.3 Laminated composite subjected to environmental conditions

Liu et al. [47] utilized an XGBoost regression model to predict the degradation of the tensile strength and tensile modulus of unidirectional FRP composites resulting from immersion in water and alkaline solutions. A database comprising 746 samples was compiled from 44 relevant experimental publications. The selection of the XGBoost model was motivated by its capability to handle missing data within the database. The model's accuracy, as measured by the coefficient of determination (R^2^), for predicting tensile strength and tensile modulus was reported as 93 % and 85 %, respectively.

##### 4.2.6.4 Framework for dataset reduction and prediction accuracy

Tao et al. [48] introduced a new methodology for enhancing ANN's accuracy and training time for the constitutive laws of unidirectional FRP composites. The technique aims to reduce the number of simulations needed for training the model by integrating ANN with the finite element analysis software ABAQUS. The proposed framework updates the ANN parameters during the simulation by updating the stiffness matrix based on ANN predictions inside a looping scheme that is controlled by the number of experimental data and specified error criteria between ABAQUS output displacements and experimentally measured displacements. The proposed framework was evaluated through two case studies: linear elastic behavior for a 3-dimensional cube under biaxial tensile loading using the SANN-MLP algorithm, comprising two hidden layers with 10 neurons each and progressive damage analysis in a 2-dimensional plate under uniaxial loading based on the Hashin failure criterion and the fracture energy dissipation using a (DNN algorithm, comprising three hidden layers with 40 neurons each. The time required for training the model on a quad-core computer was reported to be 20 min for the linear elastic problem and 10 h for the progressive damage analysis problem.

#### 4.2.7 Crack path detection

In their study, Ding et al. [28] continued investigating the utilization of Deep Learning techniques in predicting the crack path pattern in composites subjected to uniaxial tensile velocity. To this end, they compiled a dataset of 1600 crack images, which was generated through a combination of 2D FEA using the RVE model at the microscale and the discrete element model for simulating crack propagation. Their DNN model, which consisted of four hidden layers with 530 neurons in each, was found to be effective in predicting the crack path pattern, as demonstrated by its comparison with the results obtained from the FEA simulations.

#### 4.2.8 Design and optimization of FRP

##### 4.2.8.1 Stacking sequence optimization and design guidelines

Wagner et al. [49] employed ML to propose design guidelines for the stacking sequence of composite cylinders used in launch-vehicle systems for maximum buckling load and minimum sensitivity to imperfections. They generated 361 finite elements models of perfect laminated cylinders with different configurations of stacking sequence and used geometrically nonlinear analysis for perfect cylinders and lower-bound analysis to account for imperfections. They conducted a comprehensive comparative study on these models and, subsequently, focused on specific sequence structures and conducted further FEA on these types, forming their dataset samples. The DTC model was selected to establish stacking sequence guidelines for their objective in terms of non-dimensional parameters (such as bending stiffness ratio) from the classical laminate theory and used as a representation for the stacking sequence. As a verification, they established several models based on these guidelines and compared them against optimized stacking sequences proposed by other researchers, which revealed that their models remarkably outperformed the others.

Artero-Guerrero et al. [50] utilized ANN to study the effect of the stacking sequence on the ballistic limit of composites and to propose an optimum stacking sequence accordingly. An experimentally-verified finite element method (FEM) (3D) was used to generate the dataset by simulating ballistic impact on laminated CFRP composite of symmetrical 12 plies; the dataset comprised 240 samples of ballistic limit and its corresponding stacking configurations. From these datasets, SANN-MLP was trained to predict the ballistic limit with 91 % accuracy; the network structure was of one hidden layer of 9 neurons each. The ML model was then utilized for a comprehensive parametric study on different configurations of stacking sequences spanning over 2048 cases. Their ML successfully proposed a stacking sequence that increases the ballistic limit by 41 % just through the stacking sequence.

Torregrosa et al. [51] proposed a framework to be used in aeroelastic analysis instead of coupled computational fluid dynamics CFD/FEA. They implemented the SANN-MLP with the reduced-order model for the aeroelastic analysis of composite wings modeled as a single CFRP laminate bonded to a foam structure; the SANN is of 1 hidden layer with 50 neurons. Their proposed framework was trained, and the model predictions agreed well with the coupled CFD/FEA simulation. Additionally, it showed a remarkable reduction in computational costs by four orders of magnitude, which facilitated conducting a comprehensive study on the effect of the fiber direction in the CFRP on the aeroelastic response. Accordingly, they recommended that the fiber orientation within [−44°, −68°] is best for maximizing the stall flutter velocity while maintaining structural stability. Similarly, Zhang et al. [52] proposed an ML framework for stacking sequence optimization in helicopter laminated composite blades as an alternative to the traditional coupled computational fluid dynamics 3D CFD/FEM approach. Their framework utilizes principal component analysis (PCA) to extract the main characteristics of coupled CFD/FEM simulations, which is then used to train the SANN-RBF/reduced-order model. This approach reduces the computational resources required for training the framework while improving prediction accuracy. The framework was able to generate a considerable number of simulations with an average time of 35 min per simulation, compared to a corresponding FEA simulation which took 9 h per simulation. The optimized stacking sequence was obtained using a genetic algorithm on the framework-generated dataset, resulting in a 17 % reduction in maximum deflections while maintaining structural strength and stability.

The study presented by Liu et al. [53] aimed to optimize the weight of composite laminates subjected to buckling loading by reducing the number of layers and ply thickness. The researchers focused on three types of structures: flat laminate, blade-stiffened, and hat-stiffened laminates. They proposed a method that utilized ML to predict the buckling behavior of the structures as obtained from finite element analysis (FEA).To determine the optimal ML algorithm for this task, the researchers conducted a comparative study of the prediction accuracy of several models, including LR, DT, RF, and DNN. All models were trained using FEA (2D) results, with the number of simulations varying depending on the type of laminate: 750 for flat laminates, 3000 for blade-stiffened laminates, and 5000 for hat-stiffened laminates. The DNN model achieved the highest prediction accuracy in all cases and was found to have a remarkable reduction in computational cost compared to FEA, with an average time of 0.0009 % of the FEA simulation time. The DNN was then employed to generate sufficient data for the optimization problem using a genetic algorithm. The DNN network structure was of 5 hidden layers with 30 neurons for flat laminate, 5 hidden layers with 50 neurons for blade-stiffened laminate, and 6 hidden layers with 50 neurons for hat-stiffened laminate.

In a similar vein, Qiu et al. [54] employed a physically-informed CGAN for the optimal selection of fiber materials and layup stacking sequences that meet both mechanical and non-mechanical requirements, such as costs. The CGAN consisted of two neural networks: the generator and the discriminator. The researchers trained the CGAN model using 3000 FEA (3D) simulations of a composite tube under different load cases, random materials, and stacking sequences. The optimization process was achieved through the coupling of the generator and discriminator networks, where the generator proposed composite laminates that met the requirements, and the discriminator assessed the validity of the proposed designs through the learned behavior from FEA. This approach successfully suggested an optimum design and proposed a list of alternative designs.

##### 4.2.8.2 Drilling of laminated composites

Kolesnyk et al. [55] proposed a machine learning (ML) framework for predicting the hole accuracy and drilling temperature during multiple one-shot drilling of hybrid connections composed of CFRP and titanium alloy. The authors conducted one-shot drilling experiments using Taguchi orthogonal arrays, studying the effect of cutting speed, feed rate, and the time delay between drilling shots on the hole diameter/roundness variation through the hole depth and the drilling temperature variation through the hole depth. The resulting dataset consisted of 4754 samples. The authors found that a single hidden layer SANN with ten neurons achieved the best prediction metrics. In the future, they aim to use the ML framework to optimize the drilling operation.

In a related study, Zhang et al. [56] applied machine learning to predict the delamination factor from drilling parameters in laminated CFRP. The authors conducted 36 experiments using Taguchi orthogonal arrays and used a GPR model for analysis to predict the hole-exit delamination factor. The proposed model demonstrated high accuracy in comparison to experimental results.

##### 4.2.8.3 Design correction factors for composites with a hole

Freed Y [57]. conducted a study to compare the performance of various ML models in predicting the finite width correction factor for laminated composite materials with a hole. The finite width correction factor accounts for the reduction in strength observed in open-hole compression (OHC) tests when the design width-to-hole diameter ratio (W/D) is less than six or when different stacking sequences are used. The study included 24 two-dimensional FEA that were validated against published experimental OHC tests. Six different stacking sequences spanning four W/D ratios were included in the dataset, with the effective stiffness from classical laminate theory used to represent the stacking sequence. Three ML models were selected for comparison: GPR, RF, and SANN-MLP. The results showed that all three models performed similarly, and the study concluded with the presentation of carpet plots using GPR for finite width correction factors to aid in the selection of this factor in the design process.

##### 4.2.8.4 Bolted/bonded connections

Qiu et al. [58] used ML to design an even-load-distribution in bolted lap joints of CFRP. They focused on the influence of the tightening torque and hole clearance on the load distribution of a three-bolt lap joint. To generate enough data for training their ML model, they derived an equivalent electrical circuit as an analogy to the problem and established MATLAB/Simulink model, which was numerical-validated against 3D FEM. The Simulink model generated 1000 samples for training their DNN model, where a sample is a combination of hole clearance and tightening torque on each bolt and a dimensionless factor they defined as a measure of the uneven load distribution from the simulation output. Their DNN achieved high metrics in prediction with a structure of 4 hidden layers of (30,40,40,30) neurons each. They used their DNN model to conduct a comprehensive study using $2.7x10^{7}$ possible configuration of hole clearance and tightening torque, and they used genetic and particle swarm algorithms for optimization. Their study revealed that minimization of uneven-load distribution is achieved by varying the tightening torque on each bolt and smaller bolt-hole clearance at the middle bolt, which was validated experimentally.

Gajewski et al. [59] employed ML to study the strength of dual-adhesive single-lap joints under tension. The role of ML is to predict the maximum connection strength, initiation energy, and fracture energy from the geometrical parameters of the joint and the mechanical properties of the adhesives. The dataset was generated by conducting 100 FEA (3D) simulations and was used to train the SANN-MLP of 1 hidden layer with 10 neurons. The ML prediction accuracy of the three variables was approximately 98 %. Accordingly, they used the model to conduct a further correlation analysis and provide a limit curve for the adhesive parameters against fracture energy and joint strength.

The study presented by Szabelski et al. [60] used ML to accurately determine the adhesive component mix ratios above which heat holding to cure an adhesive joint will no longer significantly contribute to joint strength. The dataset was generated through experiments at ambient and elevated temperatures, with adhesive butt joints subjected to tensile loading. Their ML model was the LSTM network, which successfully predicted the mix ratio with a remarkable agreement with experimental tests.

##### 4.2.8.5 Geometrical optimization for acoustic applications

Altabey et al. [61] employed ML in their search for the optimum geometrical parameters that maximize the transmission loss factor in laminated composite mufflers. They developed a model of a dual-chamber laminated composite muffler (DCLCM) in MATLAB to generate their acoustic behavior dataset. They trained two DL networks CNN and LSTM and compared their metrics. Accordingly, CNN was then selected for its accuracy and computational speed, which was utilized to generate a large dataset for optimization using a genetic algorithm.

##### 4.2.8.6 Composite scarf repairs

Bing Yan et al. [62] examined the application of ML algorithms for designing composite scarf-bonded (CSB) structures under tensile loads. Their approach utilizes databases generated via a semi-analytical method. Adaptive Boosting (AdaBoost), Gradient Boosting Regression (GBR), Extreme Gradient Boosting (XGBoost), and Artificial Neural Networks (ANN) were utilized to predict optimal scarf angles for enhancing joint effectiveness. Among these, XGBoost is noted for its accuracy in handling complex design parameters. The models, trained on data representing various material and geometrical uncertainties, not only provide very accurate predictions of structural strength and failure modes but also efficiently map out a damage tolerance-based design space. This methodology significantly speeds up the design process by quickly estimating the most effective layup configurations for CSB structures and optimal scarf angle.

### 4.3 Statistics for ML models and sub-related topics

#### 4.3.1 Statistics for ML models

**Fig. 7:**
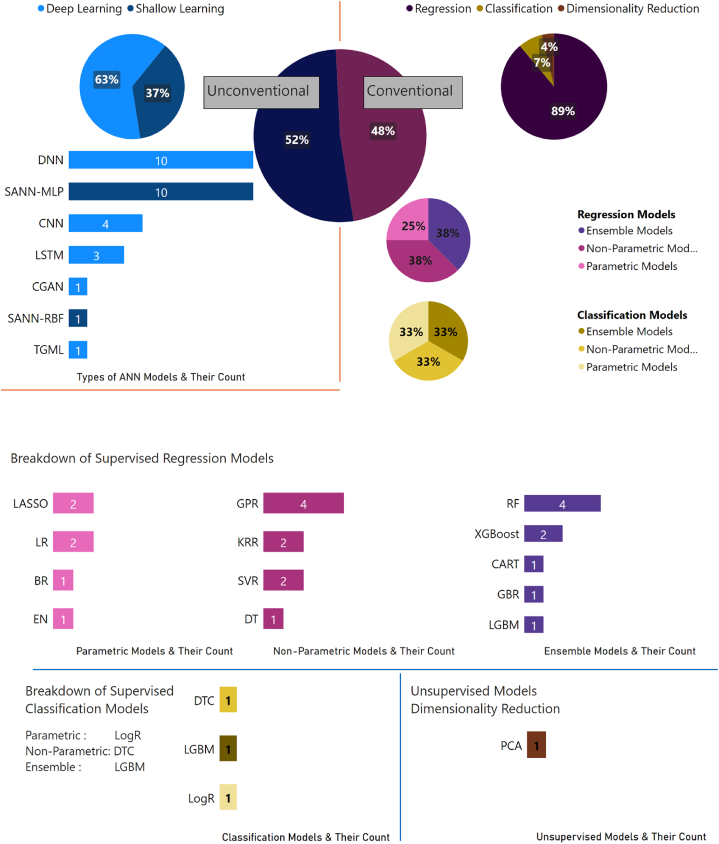
Statistics for ML algorithms used in literature.

The data presented in Fig. 7 indicates that there is a relatively even distribution of conventional and unconventional machine learning models in the literature, with 30 instances of conventional models and 28 instances of unconventional models, representing 52 % and 48 %, respectively. When further analyzing the unconventional models, it is found that shallow learning models are utilized in 11 instances, representing 37 % of the unconventional models, while deep learning models are utilized in 19 instances, representing 63 % of the unconventional models. Conventional models are shown to consist primarily of supervised regression models, which are employed in 24 instances, constituting 89 % of the conventional models. Supervised classification models are utilized in three instances, constituting 7 % of the conventional models. Additionally, there is one instance of unsupervised dimensionality reduction models, representing 4 % of the conventional models. When breaking down the supervised regression models further, it is found that there are six instances of supervised parametric regression models (25 % of supervised regression models) and nine instances of supervised non-parametric regression models (38 % of supervised regression models). Additionally, there are nine instances of supervised ensemble models (38 % of supervised regression models). With regard to specific algorithms and their usage, the SANN-MLP algorithm is utilized in ten instances, the SANN-RBF algorithm in one instance, the DNN algorithm in ten instances, the CNN algorithm in four instances, the LSTM algorithm in three instances, the CGAN algorithm in one instance, and the TGML algorithm in one instance for unconventional models. For conventional models, the GBR and CART algorithms are utilized once, the XGBoost and LASSO algorithms are utilized twice, the RF and LR algorithms are utilized four times, the LGBM, EN, BR, SVR, DT, KRR, and GPR algorithms are utilized once, the PCA and Logistic regression algorithms are utilized once, and the Decision tree classifier and LGBM classification algorithms are utilized once.

#### 4.3.2 ML libraries

**Fig. 8:**
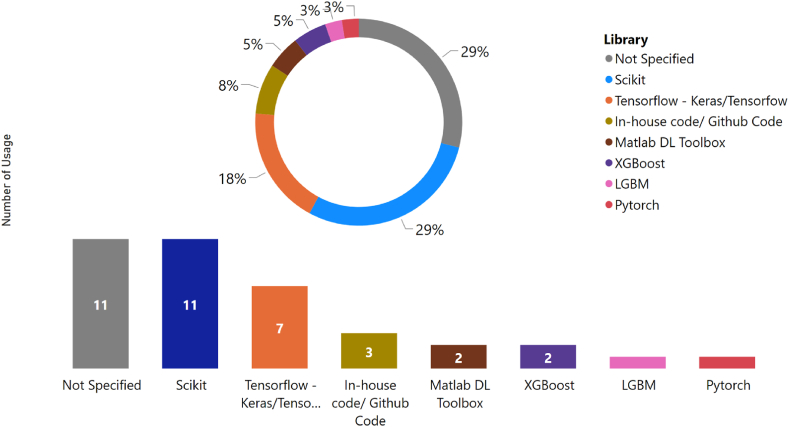
Reported ML libraries from the literature.

The libraries utilized in the literature review were recorded and presented in Fig. 8. The data depicted in the figure illustrates that the Scikit library is the most commonly used in the literature review, with 11 occurrences. Tensorflow follows as the second most frequently utilized library, with 7 occurrences. XGBoost, LGBM, Matlab DL Toolbox, in-house code, Github code, and Pytorch are also present in the literature but with a much lower frequency of usage. It is worth noting that in 11 cases, the specific library used was not specified in the literature. In accordance with the statistics of ML algorithms, it can be inferred that the Scikit Learn library is preferred for conventional ML models, while Tensorflow is more commonly used for unconventional ML models. This information can be helpful for academics when selecting appropriate libraries for their research study.

#### 4.3.3 Dataset generation

**Fig. 9:**
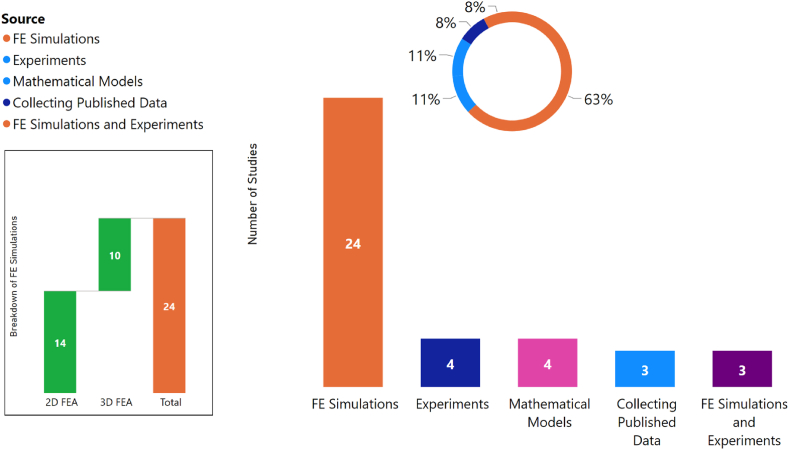
Different approaches used for establishing datasets.

A review was conducted to examine the data generation sources in the literature. The results of this review are presented in Fig. 9. The data illustrates that the most prevalent dataset generation approach is through FE simulations, with 24 occurrences. The second most common approach is the use of experiments, with 4 occurrences. Other approaches, such as the combination of FE simulations and experiments, the use of mathematical models, and the collection of published data, each have 3 occurrences. It is worth noting that a further breakdown of the use of FE simulations reveals that 2D FE simulations are more frequently used than 3D FE simulations, with 14 occurrences in comparison to 10 occurrences. This trend is particularly evident in the areas of microstructure-property linkage and damage parameter predictions and implies a preference for two-dimensional simulations in these areas.

#### 4.3.4 Impact of dataset size on ML model selection

A review was conducted to evaluate the impact of dataset size on the selection of appropriate ML models (Conventional and Unconventional). This review was based on studies that performed a comparative evaluation of the performance of various ML algorithms. The studies reviewed were those byKaramov et al. [32], Yin et al. [33], Zhang et al. [39], Post et al. [41], Liu et al. [53], and Freed Y [57]. and summarized in Table 1. Due to the high variation of the dataset size, these studies were categorized based on a dataset size limit of 1000 samples and below and 10,000 samples and above.

**Table 1:** Different outcomes from comparative studies using different dataset sizes.

	1000 Samples and below	Best Performance
Study By	Karamov et al. [32]	Conventional
Data Source	Experimentally Generated	
Study By	Liu et al. [53]	Unconventional
Data Source	FE- Generated	
Study By	Yin et al. [33]	Close Performance
Data Source	Both FEA and Experiments	
Study By	Freed Y [57].	Conventional
Data Source	FE-Generated	

	10,000 Samples and above	Best Performance
Study By	Post et al. [41]	Unconventional
Data Source	Mathematical Model	
Study By	Zhang et al. [39]	Unconventional
Data Source	FE- Generated	

For the case of comparative studies conducted using a dataset size of 1000 and below, the findings of Karamov et al. indicated that conventional models outperformed unconventional models where in their case, their dataset is generated experimentally. Liu et al. showed the contrary using an FE-generated dataset. This highlights the importance of considering the source of data generation when evaluating the performance of ML algorithms. Experimentally generated datasets are more susceptible to noise, as opposed to FE-generated datasets. This claim could be provisionally supported by the work of Yin et al. [33], whose dataset was established through a combination of FEA and experiments in which they reported a close performance between these models for a mixed dataset. The findings of Freed Y. combined with Yiu et al. show that both conventional and unconventional models be used for the FE-generated dataset below and up to 1000 samples.

However, it should be noted that these observations are provisional and should not be taken as a definitive reference due to the limited number of comparative studies conducted in this range and the complexity of the objectives of these studies. The importance of addressing this issue is to highlight that the choice of dataset size is still not settled and that different factors can affect the choice.

For the case of comparative studies conducted using a dataset size of 10,000 and above, unconventional models outperformed conventional models in this range. This observation is limited only to datasets generated through mathematical models and FEA.

## 5 Conclusion

### 5.1 Overview of machine learning applications in laminated FRP composites

The recent application of machine learning (ML) in the design and optimization of fiber-reinforced polymer (FRP) composites has been widely studied. Researchers have used various conventional and unconventional ML techniques to predict and optimize the mechanical properties and performance of FRP composites. For instance, studies have shown that ML can be used to predict the material properties of laminated composite plates and their failure behavior and to predict the delamination initiation and propagation in composite laminates. Additionally, ML has been used in design-related studies, such as optimizing the lay-up configuration of laminated composite plates for specific load cases to the design of even load distribution in bolted lap joints of CFRP. The results of the reviewed studies demonstrate ML's potential in designing and optimizing FRP composites and suggest that further research in this area could lead to significant advancements in using FRP composites in various engineering applications.

### 5.2 Adopted techniques and challenges of current ML

The supervised parametric regression models, such as the Least Absolute Shrinkage And Selection Operator (LASSO), showed a limited successful application where it is only used as an approach for reducing the number of inputs via feature selection.

Supervised non-parametric and ensemble regression models have been used in various studies to predict various properties of laminated composite materials, including strength, stiffness, and correction factors. These models have been shown to have good prediction accuracy in some cases, but they may not be able to capture the complex relationships between the inputs and outputs, as well as more advanced models such as shallow neural networks (SANN) and deep learning models. The reason for being case-specific could be due to the specific characteristics and complexity of the problem being solved. For example, the complexity of the relationships between the input and output variables and the number of input variables all play a role in determining the most appropriate model to use. Additionally, the dataset size plays a role in the choice of model, as larger datasets may be better suited for more complex models, such as deep learning models, while smaller datasets may be more appropriate for supervised regression models.

There are cases where these supervised regression models have performed better than ANNs. For example, in some studies, non-parametric models such as Gaussian Process Regression (GPR) have been shown to perform well for certain tasks, even when compared to the shallow neural network (SANN), as in Ref. [57]. Additionally, ensemble models such as Random Forest (RF) and Extreme Gradient Boosting (XGBoost) have also been shown to perform well in some cases, as they can leverage the strengths of multiple models to improve performance, as in Ref. [32]. However, it is essential to note that the performance of these models can be highly dependent on the specific case and dataset size and that the best model for a given task may need to be determined through a comprehensive evaluation.

Despite the limited appearance of supervised classification models, the non-parametric classification models, such as the Decision Tree Classifier (DTC), showed a remarkable adoption in the optimization of stacking sequence of laminated FRP, as in Ref. [49].

Deep learning models, including convolutional neural networks (CNN) and long short-term memory networks (LSTM), have been used in several studies to predict the properties of laminated composite materials and optimize geometrical parameters. These models have been shown to have high prediction accuracy and are able to capture complex relationships between inputs and outputs. Additionally, they have been used in combination with optimization algorithms to generate large datasets for optimization.

### 5.3 Lack of adopting design of experiments (DoE)

It has been noted from the literature that there is a significant lack of adoption of Design of Experiments (DoE) methodologies. Adopting DoE can dramatically reduce the size of required training datasets while maintaining or even enhancing the quality of the machine learning model outputs. This approach makes the dataset more efficient in terms of size and information richness.

### 5.4 Emphasis on interpretable AI algorithms

Implementing interpretable AI techniques, such as LIME (Local Interpretable Model-agnostic Explanations) and SHAP (SHapley Additive exPlanations), can significantly enhance the interpretability of complex models (Black-Box) like deep neural networks and ensemble methods. These techniques provide clear and comprehensible explanations of prediction outcomes, which are crucial for understanding how specific design variables contribute to the final predictions or classifications. This transparency is invaluable, particularly in fields requiring precise and verifiable predictive modeling.

### 5.5 Automated machine learning

Given the multidisciplinary nature of applying ML to the design of laminated FRP composites and considering the current number of relevant publications, researchers who lack deep expertise in machine learning can greatly benefit from the advent of automated machine learning (AutoML). AutoML presents an opportunity to optimize ML workflows, making advanced ML techniques more accessible to materials scientists. This increased accessibility is crucial for broadening the application of machine learning, enabling innovative approaches across various disciplines and applications. Such tools can facilitate ML usage, helping to overcome the steep learning curve often associated with these technologies and fostering a wider adoption in the engineering community.

## CRediT authorship contribution statement

**Sherif Samy Sorour:** Writing – review & editing, Writing – original draft, Visualization, Resources, Methodology, Investigation, Formal analysis, Conceptualization. **Chahinaz Abdelrahman Saleh:** Supervision. **Mostafa Shazly:** Supervision.

## Declaration of competing interest

The authors declare that they have no known competing financial interests or personal relationships that could have appeared to influence the work reported in this paper.

## Figures and Tables

**Fig. A1:**
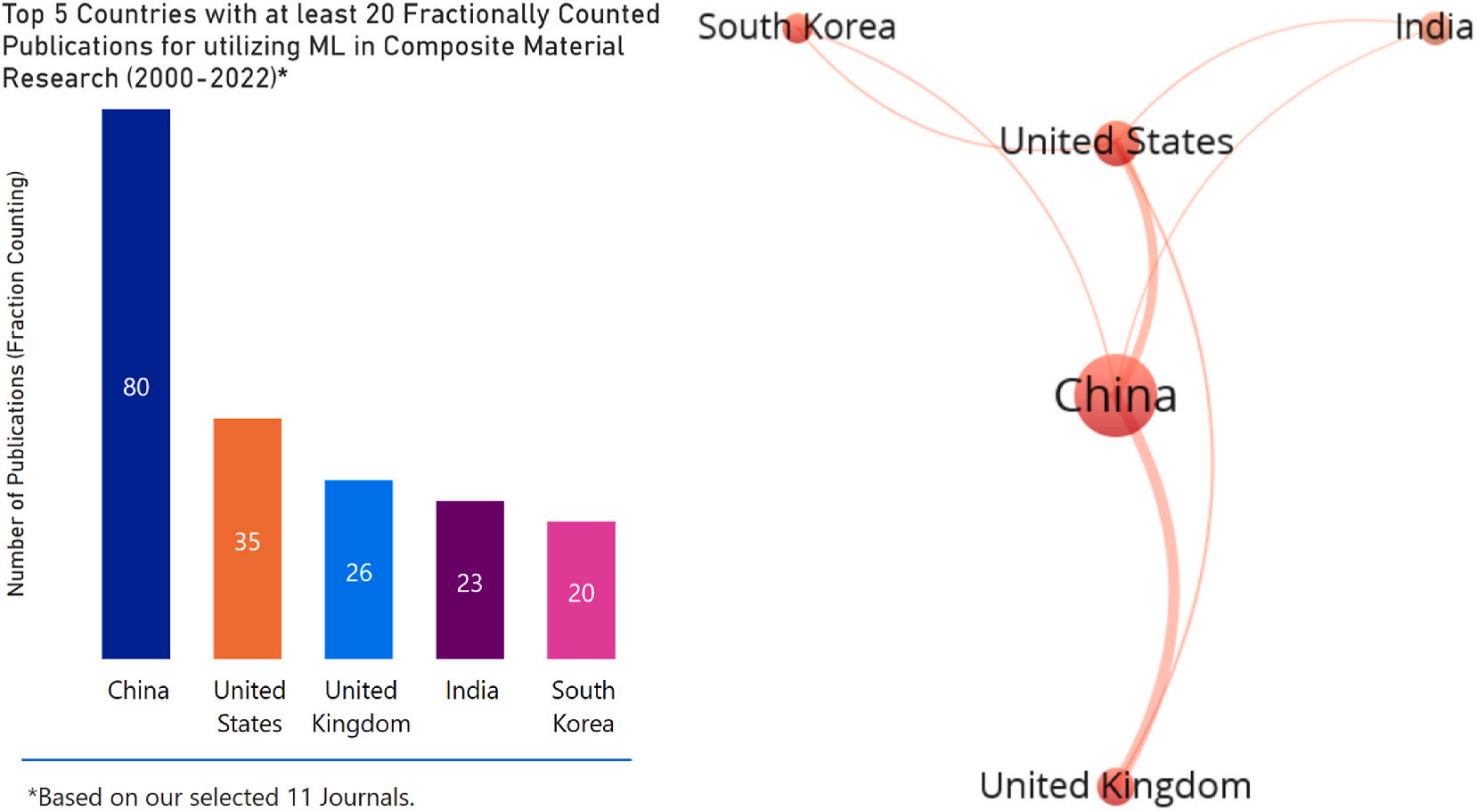
Country Rank for Implementing Machine Learning in Composite Materials Research (2000–2022) Based on the Selected Journals and Fraction Counting of Publications.

**Fig. A2:**
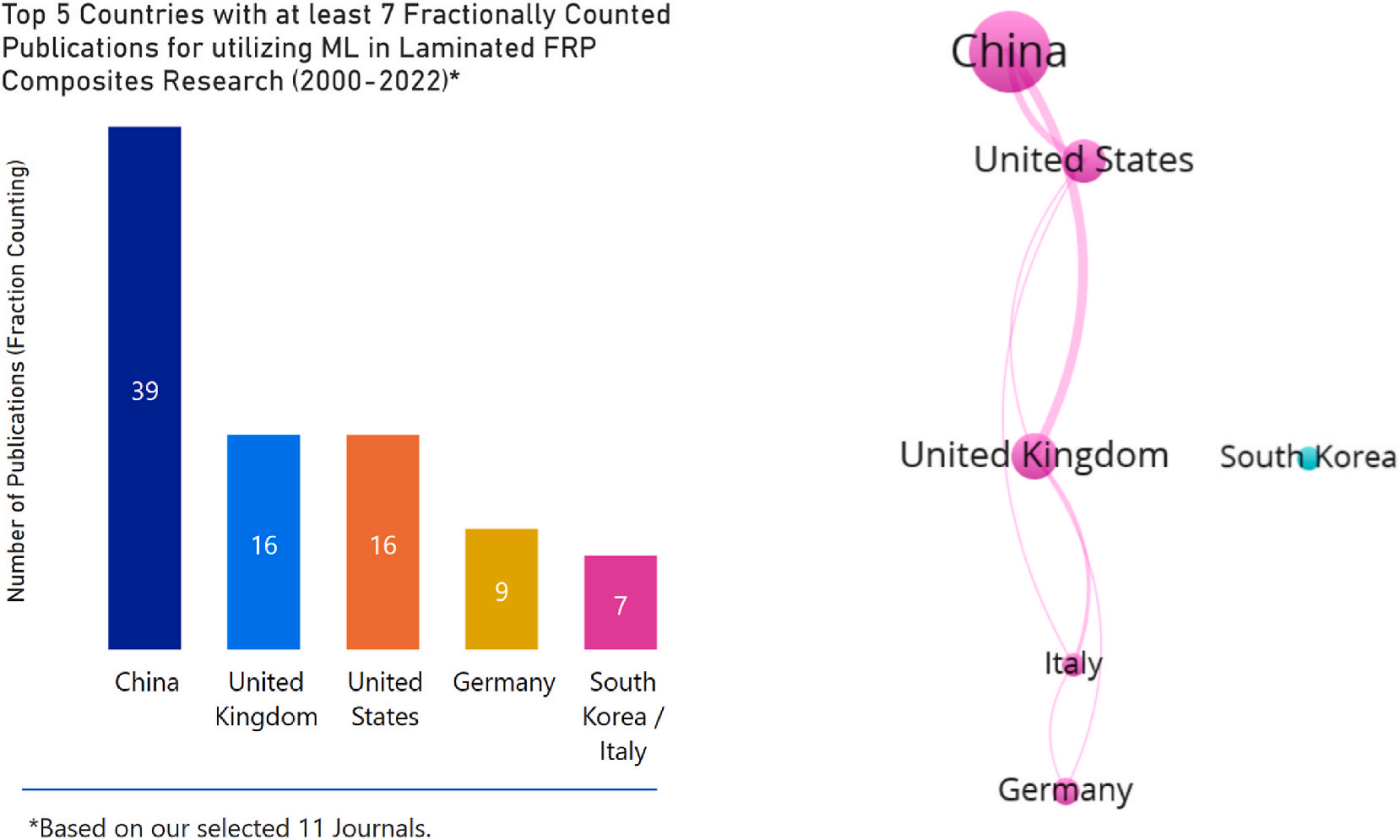
Country Rank for Implementing Machine Learning in Laminated FRP Composites (2000–2022) Based on the Selected Journals and Fraction Counting of Publications.

**Fig. A3:**
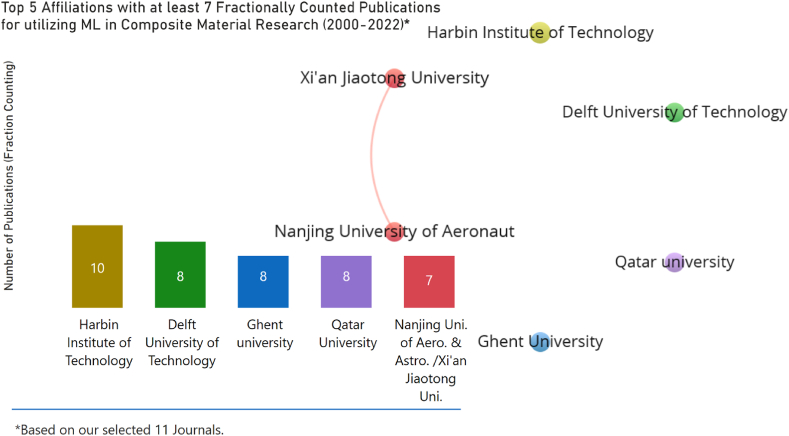
Top 5 Institutions for Implementing Machine Learning in Composite Materials Research (2000–2022) Based on the Selected Journals and Fraction Counting of Publications.

**Fig. A4:**
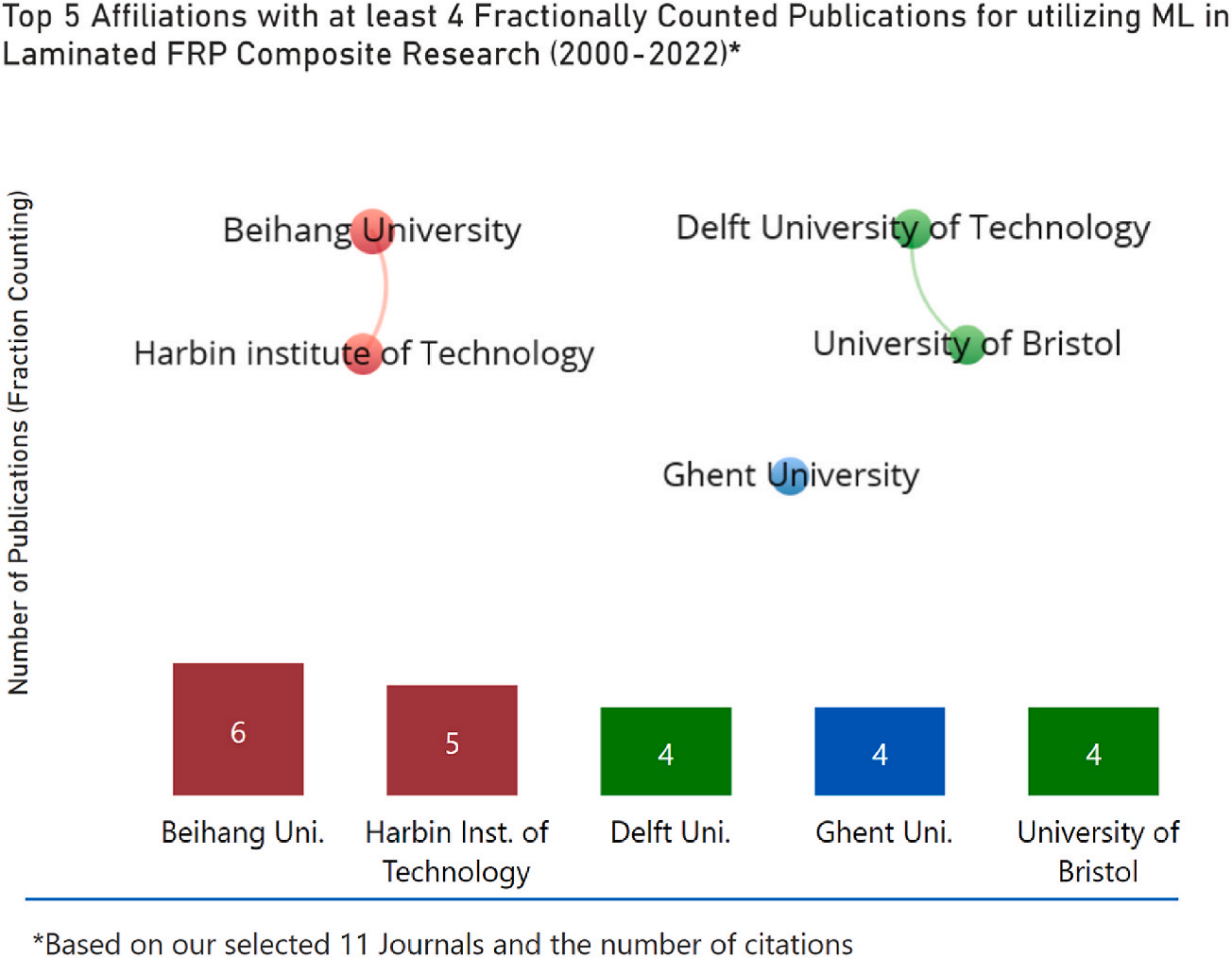
Top 5 Institutions for Implementing Machine Learning in Laminated FRP Composites (2000–2022) based on the Selected Journals and Both Fraction Counting of Publications and the Number of Citations.

## Nomenclature

AI: Artificial Intelligence
ANN: Artificial Neural Network
BR: Bayesian Ridge
CART: Classification And Regression Tree
CGAN: Conditional Generative Adversarial Network

**CNNConvolution Neural Network**
DL: Deep Learning

**DNNDeep Neural Network**
DT: Decision Tree
DTC: Decision Tree Classifier
EN: Elastic Net
FE: Finite Element
FEA: Finite Element Analysis
FRP: Fiber - Reinforced Polymer
GBR: Gradient Boosting Regressor
GNAS: Greedy Neural Network Search
GPR: Gaussian Process Regression
KRR: Kernel Ridge Regression
LASSO: Least Absolute Shrinkage And Selection Operator
LGBM: Light Gradient-Boosting Machine
LogR: Logistic Regression
LR: Linear Regression Model
LSTM: Long Short-Term Memory
ML: Machine Learning
PCA: Principal Component Analysis
PyMKS: Python Material Knowledge Systems
RF: Random Forest
RUC: Repeated Unit Cell
RVE: Representative Volume Element
SANN-MLP: Shallow Artificial Neural Network - Multi-Layer Perceptron
SANN-RBF: Shallow Artificial Neural Network - Radial Bases Function
Sfepy: Simple Finite Elements In Python
SVR: Support Vector Regression
TGML: Theory-Guided Machine Learning
XGBoost: Extreme Gradient Boosting

## Appendix (Worldwide Collaboration)

In order to construct a global and institutional collaboration map based on bibliographic data, the countries, affiliations, and publication years of the 278 papers were submitted to a mathematical network analysis software called Vosviewer [63]. Developed by Leiden University, this software is based on the VOS model proposed by CWTS researchers in the early 2000s. The collaboration map from Vosviewer can be interpreted as follows; the position of a country/institution on the bibliographic map reflects the tendency of co-authorship coupling (i.e., collaboration). Thus, the closer two countries/institution are, the more likely they appear on the same paper. The thickness of the network lines is a measure of collaboration over time; thin lines indicate collaboration occurred over a limited period or recently, while thick lines indicate that collaboration occurred at different periods, indicating a more frequent collaboration.

### A.1 Country-Level collaboration

A global contribution map was generated through fractional counting (i.e., multiple appearances of the same country per paper are counted as 1), resulting in 49 countries. To further narrow down the results and highlight the top 5 countries, a criterion of a minimum of 20 papers per country was selected. The outcome of this analysis is summarized in Fig.A1, which demonstrates that China significantly leads in contributions, followed by the United States, the United Kingdom, India, and South Korea, with relatively close contributions. In terms of frequent collaboration, a noteworthy connection between China and both the United States and the United Kingdom can be observed. A similar analysis was performed on publications in laminated FRP composites, resulting in 35 countries. To further filter the results and highlight the top 5 countries, a criterion of a minimum of 7 papers per country was selected. The outcome of this analysis is summarized in Fig.A2, which reveals a high contribution from China, a similar level of contribution from the United States and the United Kingdom, followed by Germany, and a tie between South Korea and Italy. In terms of frequent collaboration, the same notable connection between China and both the United States and the United Kingdom can be observed, and another observation is that South Korea tends to publish independently.

### A.2 Institutional-Level collaboration

The institutional contribution map generated through fractional counting yielded 341 institutions. To further narrow down the results and highlight the top 5 institutions, a criterion of a minimum of 7 papers per institution was selected. The results are presented in Fig.A3 and show relatively close contributions from the following institutions: Harbin Institute of Technology, Delft University of Technology, Ghent University, Qatar University, and a tie between the Nanjing University of Aeronautics and Astronautics and Xi'an Jiaotong University, where the only mutual collaboration is noted between these two institutions. Following the same manner for the publications in laminated FRP composites, a total of 156 institutions were obtained. To further filter the results to show the top 5 institutions, a criterion based on a minimum of 4 papers per institution and an additional criterion based on the number of citations are selected due to the large pool of institutions of similar fraction counting. The outcome of this analysis is illustrated in Fig.A4, and it reveals a relatively equal contribution for the following institutions; Beihang University, Harbin Inst. of Technology, Delft University, University of Bristol, and Ghent University. In terms of collaboration, two clusters of research can be noticed, a mutual collaboration between Beihang University and Harbin Inst. of Technology and, similarly, between Delft University and the University of Bristol.
